# Two Types of Functionally Distinct Fiber Containing Structural Protein Complexes Are Produced during Infection of Adenovirus Serotype 5

**DOI:** 10.1371/journal.pone.0117976

**Published:** 2015-02-27

**Authors:** Bo Zhang, Yuhua Yan, Jie Jin, Hongyu Lin, Zongyi Li, Xiaoyan Zhang, Jin Liu, Chao Xi, Andre Lieber, Xiaolong Fan, Liang Ran

**Affiliations:** 1 Beijing Key Laboratory of Gene Resources and Molecular Development, College of Life Science, Beijing Normal University, Beijing, China; 2 Division of Medical Genetics, University of Washington, Seattle, Washington, United States of America; University Claude Bernard Lyon 1, FRANCE

## Abstract

Adenoviruses are common pathogens. The localization of their receptors coxsackievirus and adenovirus receptor, and desmoglein-2 in cell-cell junction complexes between polarized epithelial cells represents a major challenge for adenovirus infection from the apical surface. Structural proteins including hexon, penton base and fiber are excessively produced in serotype 5 adenovirus (Ad5)-infected cells. We have characterized the composition of structural protein complexes released from Ad5 infected cells and their capacity in remodeling cell-cell junction complexes. Using T84 cells as a model for polarized epithelium, we have studied the effect of Ad5 structural protein complexes in remodeling cell-cell junctions in polarized epithelium. The initial Ad5 infection in T84 cell culture was inefficient. However, progressive distortion of cell-cell junction in association with fiber release was evident during progression of Ad5 infection. Incubation of T84 cell cultures with virion-free supernatant from Ad5 infected culture resulted in distortion of cell-cell junctions and decreased infectivity of Ad5-GFP vector. We used gel filtration chromatography to fractionate fiber containing virion–free supernatant from Ad5 infected culture supernatant. Fiber containing fractions were further characterized for their capacity to inhibit the infection of Ad5-GFP vector, their composition in adenovirus structural proteins using western blot and LC-MS/MS and their capacity in remolding cell-cell junctions. Fiber molecules in complexes containing penton base and hexon, or mainly hexon were identified. Only the fiber complexes with relatively high content of penton base, but not the fiber-hexon complexes with low penton base, were able to penetrate into T84 cells and cause distortion of cell-cell junctions. Our findings suggest that these two types of fiber complexes may play different roles in adenoviral infection.

## Introduction

Human adenoviruses (HAdVs) are nonenveloped viruses causing a variety of infections in respiratory, ocular and enteric systems. Fifty-one types of HAdVs based on serum neutralization and 17 additional based on genome sequencing and bioinformatics analysis classified into A to F subgroups, are currently identified [1]. The adenoviral capsid is composed of three major structural proteins: hexon, penton base and fiber. The infection of host cells by HAdVs is initiated by fiber binding to its receptors on the surface of host cells. *In vitro* studies have demonstrated that the majority of HAdVs utilize coxsackievirus and adenovirus receptor (CAR) as a receptor to initiate the infection [2]. HAdVs of subgroup B utilize either CD46 [3,4], or Desmoglein 2 (DSG-2) [5] to initiate infection. Ad37 (subgroup D), a leading cause of epidemic keratoconjunctivitis, utilizes GD1a glycan [6]. The binding of fiber molecules to receptors mediates the attachment of viral particle to host cells, which then is followed by penton base-mediated interactions with integrin molecules for internalization of adenoviral particles [7].

CAR molecules, as well as DSG-2 are confined to the cell-cell junction complexes in polarized epithelium [5,8]. The initial round of HAdVs infection and HAdVs vector-mediated gene transfer of polarized epithelium is thus expected to be inefficient. CAR is a component of the tight junction (TJ) between polarized epithelial cells [9–11]. TJs are the most apical cell-cell junctions of the epithelium [12], which generate an impermeable barrier between the epithelium and the extracellular environment, and seal the space between neighboring cells. CAR molecules are thus inaccessible to HAdVs approaching from the apical surface. CAR contains a single transmembrane domain that separates its two IgG-like extracellular domains from an intracellular domain with PDZ-binding motif. The D1 extracellular domains of CAR molecules of the adjacent cells form homophilic interactions within the structure of cell-cell junction. However, the weakly expressed CAR isoform, CAR^Ex8^, was reported to localize to the apical membrane of epithelia [13]. The intracellular domain of the CAR plays a crucial role in the confined localization of CAR to TJ, because glycophosphatidylinositol-anchored CAR mutant as well as CAR mutant lacking the intracellular tail was found to be diffusely localized on the apical plasma membrane [9]. The intracellular domain of CAR also mediates the interaction with another TJ component, zona occludens-1 (ZO-1) that provides the link with the actin cytoskeleton [14].

In the course of HAdV infection, fiber and penton base are excessively produced relative to the need of Ad particle assembly [15–17]. Fiber is also present on defective viral particles, which are produced in excess in Ad infected cells. Overproduced fiber and penton base from Ad3, Ad7, Ad11 and Ad14 form dodecahedral particles [5,18–20]. The fiber-containing Ad3 dodecahedra can efficiently attach to cells and penetrate them [18,21]. However, Ad5 infection is known not to form such dodecahedra [18]. Previous studies suggested that overproduced fiber molecules facilitate the assembly of progeny viral particles [17]. In non-polarized cells, we previously demonstrated that fiber molecules, released from Ad infected cells, cause receptor masking in non-infected bystander cells, thereby inhibiting these cells from infection by progeny viruses [16]. In studies on polarized epithelial cell cultures, fiber knob molecules, applied from the basolateral surface, were demonstrated capable of opening cell-cell junctions by competitive binding to CAR, suggesting that fiber molecules excessively produced during HAdV infection can promote the spread of progeny viruses [22]. However, it is currently unknown whether free fiber molecules function alone, or in concert with other structural proteins in remodeling cell-cell junction complexes.

Ad5 is one of the most extensively characterized HAdVs. In this report, we examined the effect of Ad5 fiber-containing complexes during infection of polarized T84 epithelial cells. Fiber molecules in complexes with penton base and hexon, or predominantly with hexon were identified. These two types of complexes are associated with distinct functions regarding intracellular internalization following receptor binding and remodeling of cell-cell junction, thus they may play different roles during the progression of adenovirus infection.

## Results

### Fiber release and progressive distortion of cell-cell junctions during Ad5 infection of T84 cells from apical surface

**Fig 1:**
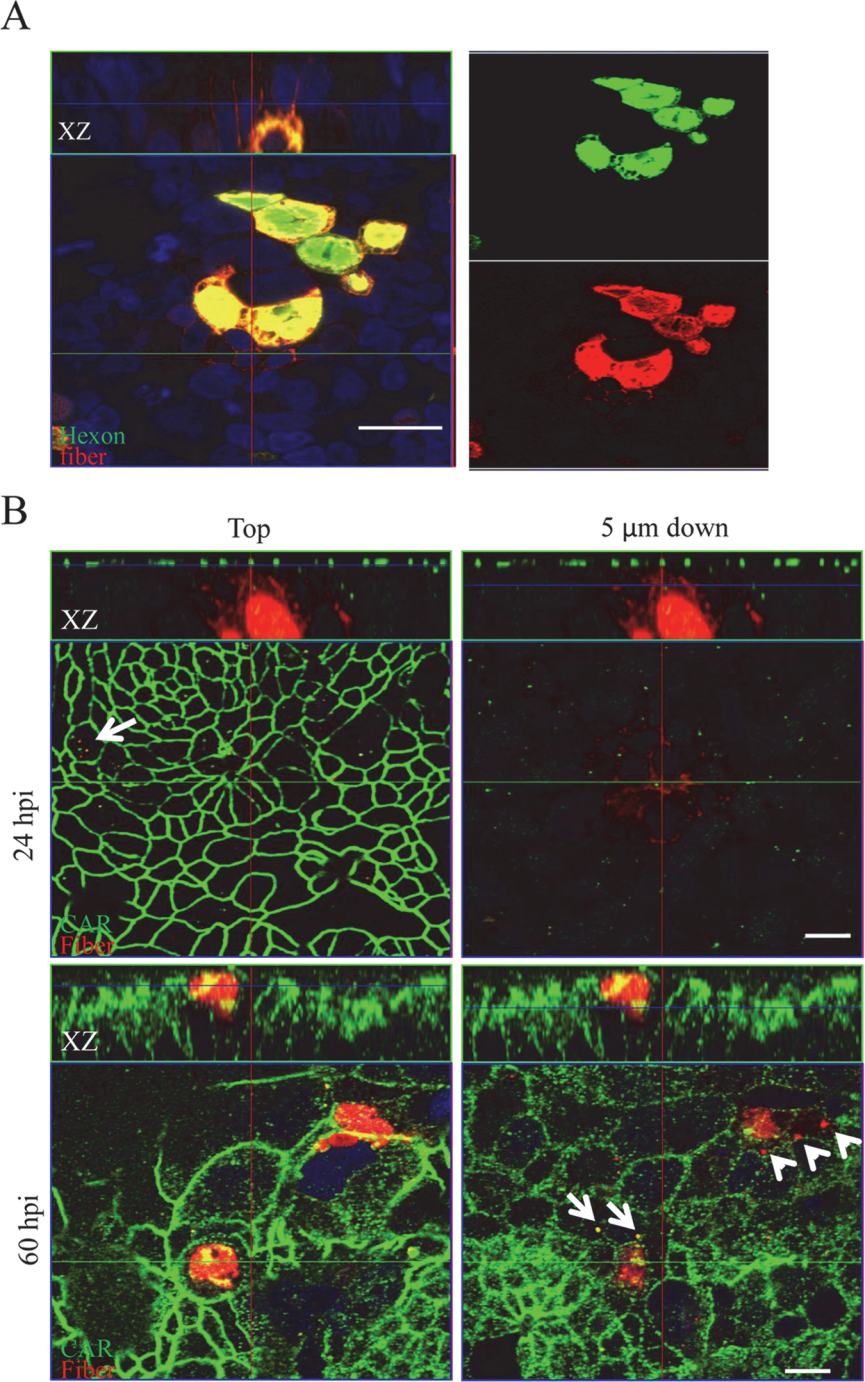
Fiber is released and TJs exhibit progressive distortion over time after Ad5 infection of polarized T84 cultures from the apical surface. (A) Fiber molecules were already released prior to cell lysis from infected cells. T84 cells cultured on transwell inserts with well-developed TJs were infected with Ad5 at a MOI of 20 pfu/cell from the apical surface. Cultures were washed twice with the DMEM-F12 medium at 4 hrs post infection. Merged and separated images of hexon (green) and fiber (red) staining show that at 30 hpi, fiber molecules were released from infected cells positively stained with hexon and bound to non-infected bystander cells. Bar: 20 μm. (B) Progressive distortion of TJs. At 30 hpi, CAR staining (green) was predominantly localized within distinct TJs at the apex of T84 cell layer. At 60 hpi, distorted staining pattern of CAR was detected at the apex, basolateral membrane and in cytoplasma of T84 cells. Internalized fiber molecules (red dots, arrow heads) and co-localization of internalized fiber and CAR (yellow dots, arrows) in non-infected cells are indicated. Bar: 10 μm. Cells were visualized using Zeiss confocal microscopy LSM510. Images are representative of multiple experiments performed in this project. The crosshairs in the plots indicate the positions for the XZ plane.

The epithelial colon cancer T84 cell line is an *in vitro* model system that enables the study of epithelial barrier regulation. We cultured T84 cells on transwell insert for at least one week until the formation of well-developed TJ, i.e. at time points when transepithelial electrical resistance was constant and above 3 000 Ω cm^2^. To study the alteration of cell-cell junctions along the progression of Ad5 infection, these cultures were then infected with wild type Ad5 from the apical surface at a MOI of 20 pfu/cell. At 30 hr post infection (hpi), only few infected cells were detected by hexon staining (Fig. 1A). However, the adjacent cells with no intracellular hexon staining showed strong fiber staining on the cell membrane (Fig. 1A). Spreading of fiber molecules from the infected, yet intact, cells were observed in both XY and XZ planes. Fiber molecules are expected to bind to CAR, which is a component of TJ complexes. We then investigated the localization and arrangement of CAR during the progression of the apical Ad5 infection of T84 cell cultures. At 30 hpi, CAR predominantly localized within TJs of the most apical regions, CAR staining was almost not detectable at 5 μm below the apical surface (Fig. 1B). Weak, however discernible internalization of CAR and fiber, and their co-localization in the cytoplasma were detected in a small fraction of the non-infected cells. At 60 hpi, the number of infected cells was still limited (Fig. 1B). However, CAR staining was not restricted to the apical surface, but reached deeper layers below the apical surface (Fig. 1B, XZ plane). Further, the pattern of CAR localization became significantly distorted at 60 hpi (Fig. 1B). The spread of fiber (red dots) and the intracellular fiber-CAR co-localization (yellow dots) were markedly more profound compared to that at 30 hpi. These data together show progressive distortion of cell-cell junctions and spread of fiber molecules to non-infected cells even though Ad5 infection initiated from the apical surface was not efficient in T84 cell cultures.

### Effect of virion-free supernatant in distortion of cell-cell junctions

**Fig 2:**
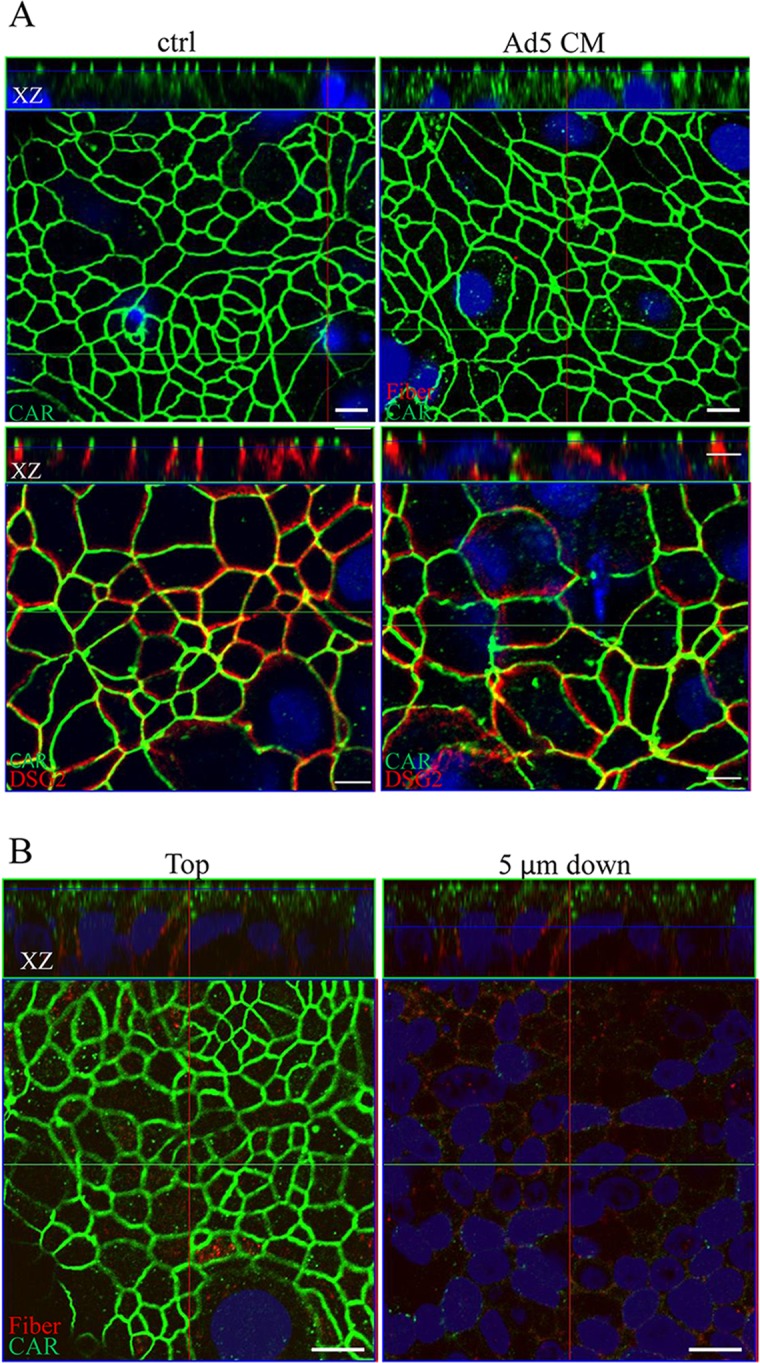
Remodeling of cell-cell junction between T84 epithelial cells by virion-free supernatant from Ad5 infected culture. Virion-free supernatant from Ad5-infected A549 culture at day 3 post infection with Ad5 at a MOI of 1 were prepared as conditioned media (CM) by ultra-centrifugation at 108 000 g. (A) T84 cells cultured on the insert of transwell until the development of TJ were apically treated with 100 μl Ad5 CM (corresponding to 4 functional units of fiber molecules) for 6 hr. CAR and DSG-2 were immuno-labeled with rabbit anti-CAR antibody and mouse anti-DSG-2 antibody followed by Alexa Flour 488 conjugated donkey anti-rabbit IgG and Alexa Flour 568 conjugated donkey-anti mouse IgG. The localization of CAR (green) and DSG-2 (red) in the control (ctrl, treated with supernatant from non-infected A549 cultures prepared in parallel to the CM from Ad5 infected A549 cultures) and Ad5 CM-treated T84 cultures are documented. Data in the XZ planes show the rearrangement of CAR and DSG2 localization. (B) T84 cells cultured as in (A) were basolaterally treated with Ad5 CM for 6 hr. Data on XY plane show diffuse CAR staining at cell-cell junction and cytoplasmic staining of CAR, as well as fiber staining following supernantant treatment; data on XZ plane show the apex-localization of CAR. Internalization of CAR and fiber molecules were found following either apical or basolateral application of Ad5 CM. Cells were visualized using Zeiss confocal microscopy LSM510. Bar: 10 μm.

To identify the component released from the infected cells responsible for the distortion of cell-cell junctions, virion-free supernatant of A549 cultures at day 3 post infection with Ad5 at a MOI of 1 (conditioned media, CM) was prepared by depletion of viral particles using ultra-centrifugation at 108 000 g (17). The CM was functionally titrated for their capacity to inhibit the infection of Ad5-GFP vector in A549 cells. In the control cultures, CAR is localized in the apex part of TJ between T84 cells, DSG-2 is below the CAR molecules with sharp and distinct staining pattern (Fig. 2A, lower left panel, XZ plane). Incubation of T84 cells with the CM from apical surface for 6 hrs resulted in dispersed CAR localization to deeper layers below the apical surface (Fig. 2A, upper right panel, XZ plane) as well as internalization of CAR (Fig. 2A, upper right panel, XY plane). The co-staining of CAR and DSG-2 further supports the distortion of cell-cell junction (Fig. 2A, lower right panel). Compared with the control cultures, DSG-2 staining became diffuse and distorted following the treatment with CM. For the same period of incubation, application of the CM from the basolateral side resulted in more pronounced distortion of CAR localization compared to that following application of CM from the apical surface (Fig. 2B). Fiber proteins were obviously bound to the cell membrane at lower basolateral side (Fig. 2B).

To test the consequence of the distortion of cell-cell junctions on Ad infection, we first applied the CM to the apical surface of T84 cultures, the cultures were then infected with replication-deficient Ad5-GFP or Ad5F35-GFP vector at a MOI 200 from the apical surface. At 48 hpi, the infection efficiency was detected by visualizing GFP-positive cells using fluorescence microscopy. In agreement with our previous report on the effect of fiber mediated receptor masking, treatment with CM from Ad5 infected cultures strongly inhibited Ad5-GFP infection [16].

**Fig 3:**
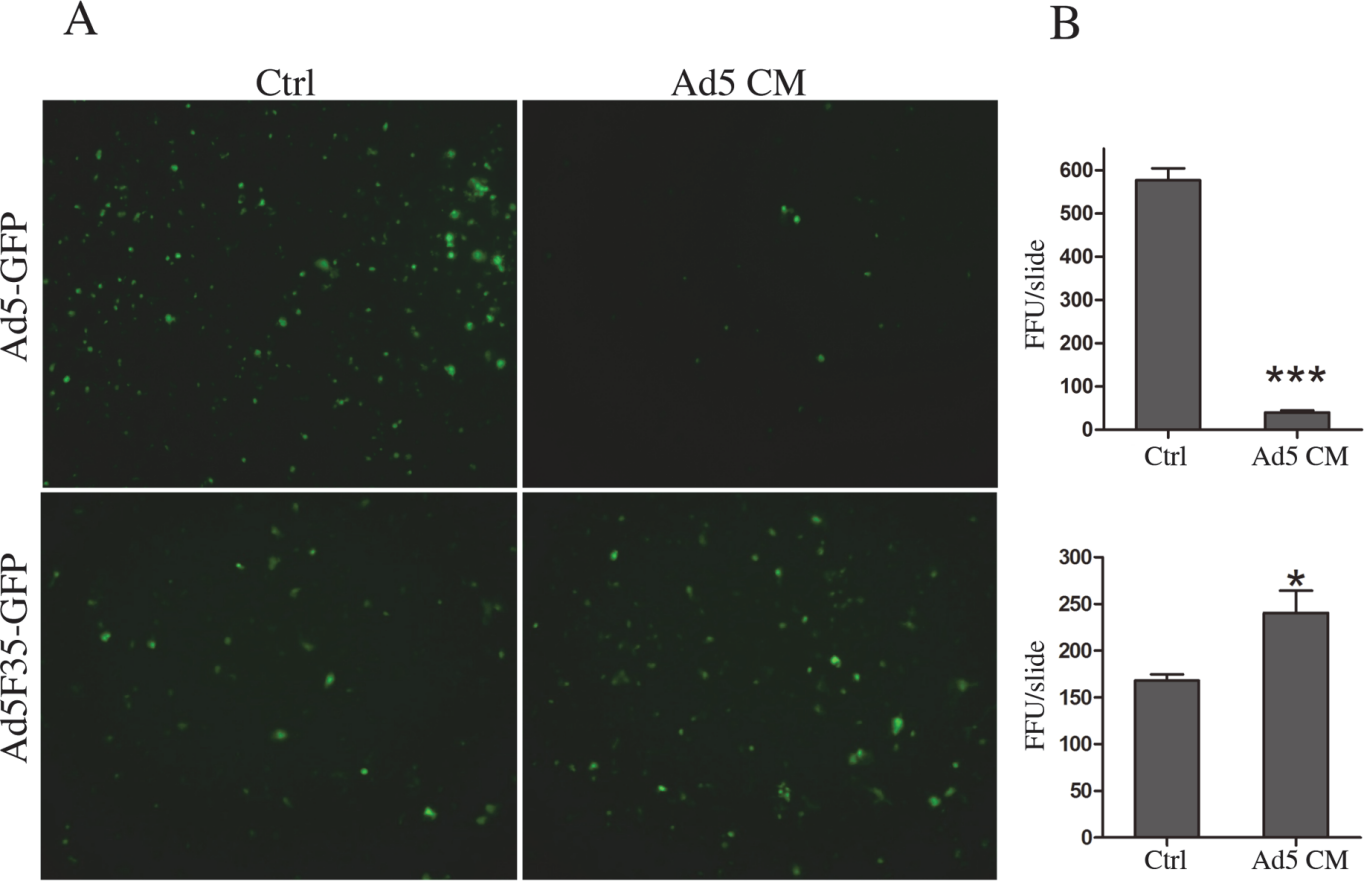
Consequences of cell-cell junction remodeling on Ad infection. (A) T84 cultures with well-developed cell-cell junctions were pre-treated with Ad5 CM (corresponding to 4 functional units of fiber molecules) or with supernatant from mock infected culture from the apical surface for 6 hr, and subsequently infected with Ad5-GFP or Ad5F35-GFP at a MOI of 200 and 100, respectively. The infected cells were visualized by GFP at 48 hpi. Pre-treatment with Ad5 CM inhibited the infection of Ad5-GFP but promoted the infection of Ad5F35-GFP. Images were taken using an Olympus IX70 microscopy with a 20x objective. (B) Average fluorescent focus forming units (FFU) per slide of 6 individual photos as shown in (A). Each treatment was preformed in triplicate and 2 photos were taken from each trial. The FFU were analyzed and calculated by ImageJ program (n = 6, *: P < 0.05, ***: P < 0.001, *t*-test). The data shown are representative of three experiments performed.

In contrast, the Ad5F35-GFP vector showed a higher infection efficiency in cultures treated with CM compared to the control cultures (Fig. 3). This finding suggested that distortion of cell-cell junctions was associated with enhanced accessibility to CD46 receptors, which is consistent with previous report that CD46 is often entrapped in tumoral cell junctions such as in the BT474 breast cancer cell line. Interestingly, addition of Ad3 dodecahedron (Dd) to these cells enhanced their transduction by the Ad5-F35-betaGal vector, suggesting that a Dd-triggered cell remodeling made CD46 more accessible to Ad5F35 vector [5].

### Two types of functionally distinct fiber complexes in virion-free supernatant

**Fig 4:**
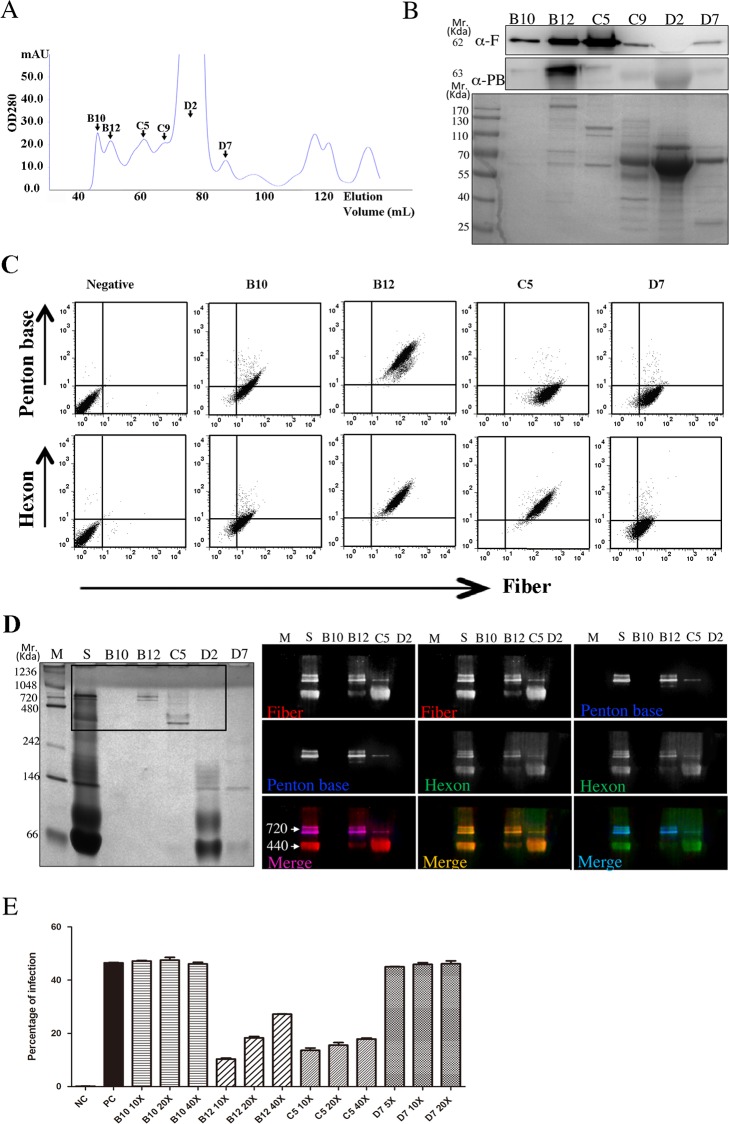
Identification and characterization of structure protein complexes in the Ad5 CM. (A) Elution profile of gel filtration chromatography. One ml of concentrated Ad5 CM was separated by Superdex 200 column at a flow rate of 0.5 ml/min. Protein concentration of each fraction was monitored with absorbance at 280 nm (Y axis). The indicated fractions (arrow), named according to their positions on the collection rack, were selected for the analyses performed in panels B to E. (B) Upper panel: western-blot analysis for the detection of fiber (F) and penton base (PB) in the fractions from (A); lower panel: SimplyBlue staining in the corresponding SDS-PAGE gel. Fiber molecules were abundant in B12 and C5 fractions, penton base mainly in B12 fraction. (C) Identification of Ad structure proteins in fiber-containing fractions by cell surface binding assay. A549 cells were incubated with 200 μl of the indicated fractions for 15 min on ice. Cells were then washed and incubated with the 4D2 anti-fiber mAb in combination with rabbit anti-penton base antibody, followed by staining with Alexa Flour 647 conjugated goat anti-mouse IgG and Alexa Flour 488 conjugated goat anti-rabbit antibody. Parallel samples stained with 4D2 mAb in combination with FITC-conjugated goat anti-hexon antibody were then stained with Alexa Flour 647 conjugated goat anti-mouse IgG. The cells were analyzed in FACS Calibur for the double stained cells. The dot-plots shown are representative of three independent experiments. Fiber, penton base and hexon were detected in B12 fraction, whereas only hexon and fiber were detected in C5 fraction. (D) Characterization of structural protein complexes by native PAGE and western-blot analysis. Proteins in different fractions were separated by pre-casted 4–20% native PAGE gel and blotted onto PVDF membrane. The membrane was probed with anti-fiber, anti-penton base and anti-hexon antibodies simultaneously and then further probed with the secondary antibodies used in (C). The membrane was scanned by a PharosFX Plus system (Bio-Rad) using 3-laser channels for the visualization of specific binding bands for different antigens. SimplyBlue protein staining of the native PAGE gel (left panel) depicts that B12 and C5 fraction contain protein complexes of about 720 kDa and 440 kDa, respectively. The area on the gel corresponding to the western-blot data shown in the right panel is indicated by the rectangle. Fiber, hexon and penton base were detected in the B12 fraction, whereas fiber and hexon were detected in the C5 fraction. (E) Capacity of fiber containing fractions in inhibition of Ad5-GFP infection in A549 cells. The fractions containing fiber molecule (B10, B12, C5 and D7) were first concentrated by a factor of 5 and subsequently diluted as indicated with fresh cell culture media, 200 μl of each dilution were applied to each well of A549 cells cultured in 24-well plates for 30 min at 37°C. Following two washes with fresh media, cells were infected with Ad5-GFP virus at a MOI of 100 for 1 hr at 37°C. The percentage of infected cells was analyzed with FACS assay as GFP positive cells at 24 hpi. NC: negative control samples (mock infection); PC: positive control samples (infection without pre-treatment with Ad5 CM).

In addition to fiber molecules, the CM from Ad5 infected culture might contain other molecules such as cytokines that could activate T84 cells, resulting in distortion of cell-cell junction. To test these probabilities, we have utilized gel filtration chromatography to separate large protein molecules from low molecular weight cytokines in the virion-free supernatant (Fig. 4A). We have also performed a control chromatography with the supernatant from Ad-GFP infected A549 cultures. Since Ad-GFP is replication deficient, Ad structural proteins are not produced. The peaks including fractions B10 to C5 as depicted in Fig 4A were not found (S1 Fig.), indicating that the absence of viral protein production may contribute significantly to the profile of protein peaks. Western-blot analysis detected high concentrations of fiber molecules in fractions between B12 and C5 (Fig. 4B). High concentration of penton base was detected in B12, but not in other fractions. To further characterize the composition of Ad structural proteins in those fiber-containing fractions, we incubated A549 cells with the same volume of the fraction B10, B12, C5 and D7 on ice, and detected the binding of Ad structural proteins to cell surface. Consistent with the results in the western-blot analysis, high intensity of fiber binding was detected in B12 and C5 fractions, and penton base was only detected in the B12 fraction. Interesting, both B12 and C5 fractions contained high concentration of hexon. Whereas in B10 and D7 fractions, weak fiber binding, no binding of penton base and hexon was detected (Fig. 4C). Next, we first used native 4–20% gradient PAGE in combination with western-blot to analyze if fiber, penton base and hexon molecules resided in the same protein complexes. In the B12 fraction, a large protein complex at around 720 kDa was observed, this complex contained fiber, penton base and hexon. However, a much smaller protein complex (at about 440 kDa) was observed in the C5 fraction; western-blot analysis showed that this complex contained fiber and hexon molecules, penton base molecules were not detectable in this fraction (Fig. 4D, right panel). Further, the main protein band of B12 and C5 fraction following the native PAGE gel separation were excised, trypsin digested and analyzed in LC-MS/MS. Penton base, fiber, adenoviral 100 kDa protein molecules (supporting hexon folding and trimerization), and hexon were identified in the protein band of fraction B12 (Table 1), whereas hexon, adenoviral 100 kDa protein, the chaperon protein heat shock protein (HSP) 90 alpha, fiber, and penton base were found in the protein band of fraction C5 (Table 1). The presence of 100 kDa protein and HSP 90 indicates that the protein complexes in both B12 and C5 fractions are formed in the presence of the accessory proteins supporting the folding of structural proteins, the trimerization of hexon proteins and the packaging of viral particles [23,24]. Fractions B12 and C5, but not other fractions including D7 containing low amount of fiber (Fig. 4B), were able to inhibit Ad5-GFP infection in A549 cells at high dilutions (Fig. 4E).

**Table 1:** LC-MS/MS identification of proteins in the B12 and C5 fraction.

Fraction	Protein	Mascot score	Mr	Matched peptides (p < 0.05)	Total protein coverage
B12	penton base	3643	63482	81	54%
	fiber	3070	61475	65	48%
	100 kDa protein (Hexon associated protein of Ad-C)	618	91411	20	41%
	Single strand DNA binding protein	242	59790	5	23%
	hexon	135	108338	5	8%
					
C5	Hexon	6954	108303	179	54%
	100 kDa protein (Hexon associated protein of Ad-C)	600	91411	19	31%
	heat shock protein 90-alpha	253	85006	10	20%
	fiber	211	11915	3	32%
	penton base	185	63682	4	16%
	VI capsid protein	112	27054	2	24%

The main protein band of the B12 and C5 fraction as shown in Fig. 4D was excised from the native PAGE gel following staining with SimplyBlue (Invitrogen). The proteins were digested with trypsin, the resulting peptides were separated by capillary liquid chromatography/tandam mass spectrometry using ESI-QUAD-TOF. The identity of the peptides was searched for in the NCBI protein database for human adenoviruses. The percentages of total protein coverage indicate the proportion of peptide sequences identified in this LC-MS/MS analysis in relation to the complete sequences of a given protein, it did not correspond to the content of the proteins in the B12 or C5 fraction.

**Fig 5:**
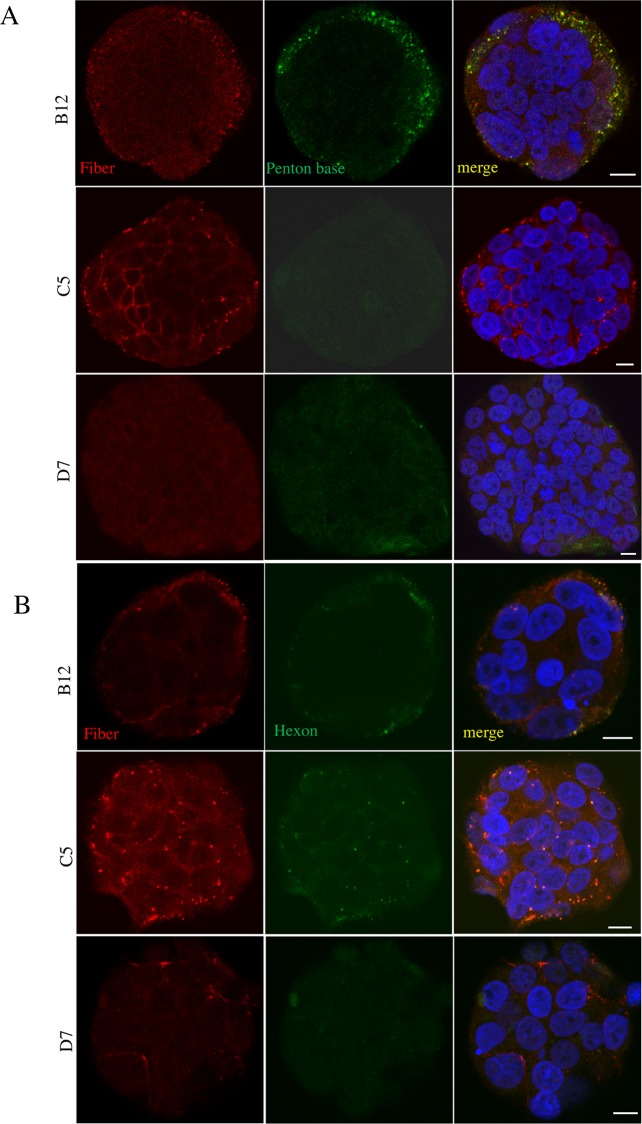
Only penton base-containing fiber complexes can penetrate into host cells. T84 cells cultured on 8-well chamber slides were incubated with protein fractions B12, C5 and D7 at 5x dilutions in fresh media for 1h at 37°C. Cells were then fixed, permeabilized and double labeled with anti-fiber and anti-penton base antibodies or with anti-fiber and anti-hexon antibodies. (A) Data from co-staining of penton base and fiber. (B) Data from co-staining of fiber and hexon. Fiber, penton base and hexon were co-localized intracellularly in cells incubated with fraction B12. Fiber molecule clearly labeled the cell-cell borders after incubation with fraction C5. The nuclei were stained with DAPI using Vectachield mounting medium. Cells were visualized using Zeiss confocal microscopy LSM700. Data shown are representative of the results with the fractions from three independent gel filtration chromatography experiments.

**Fig 6:**
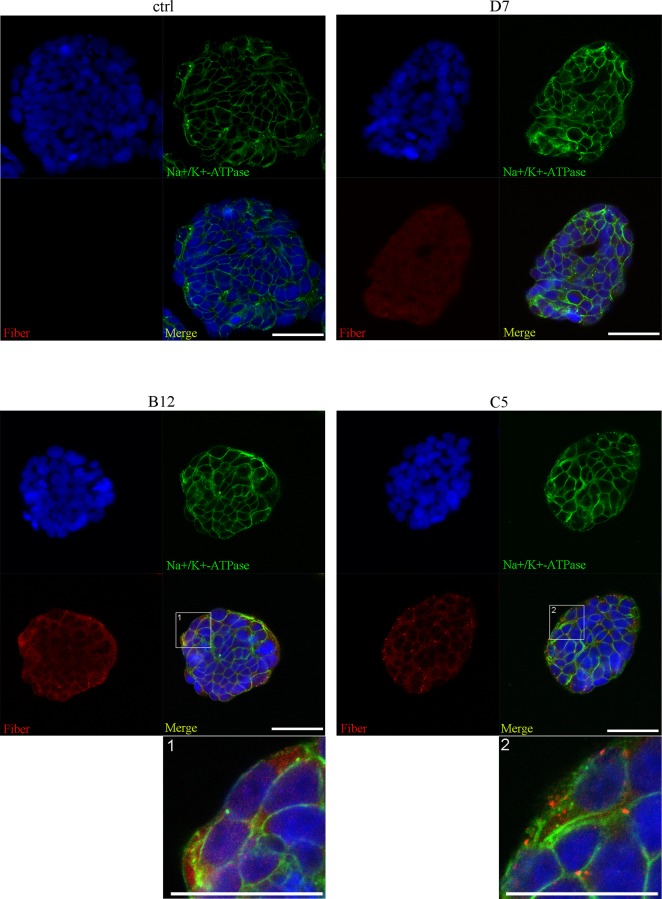
Only penton base-containing fiber complexes can penetrate into host cells. T84 cells cultured and treated as in Fig. 5 were co-stained with fiber and sodium potassium ATPase. The staining results were visualized using the same conditions as in Fig. 5. Fiber molecules were found to be predominantly intracellularly following B12 treatment.

Thus, fiber molecules are present in two types of complexes: complexes predominantly containing fiber and hexon molecules, and complexes containing fiber, penton base and hexon molecules. Both types of complexes are expected to bind to CAR on host cell surface. To study their fate following binding to CAR, we incubated T84 cells cultured on cover glass with B12 or C5 fraction for 1 hr at 37°C. Dispersed co-localization of fiber and penton base was observed in T84 cells incubated with fraction B12 (Fig. 5). In contrast, fiber and hexon molecules from fraction C5 apparently labeled the border between the cells, without apparent internalization. Further, co-staining of fiber and sodium potassium ATPase (a marker of plasma membrane) showed that following B12 treatment, fiber molecules were found to be predominantly intracellular (Fig. 6). In the T84 cultures treated with C5 fraction, fiber molecules were however mainly located at the plasma membrane. Weak fiber binding was detected following incubation with D7 fraction. These findings suggest that penton base is crucial for internalization of fiber complexes; and fiber-containing complexes without the support of penton base can bind to CAR, but not be internalized.

**Fig 7:**
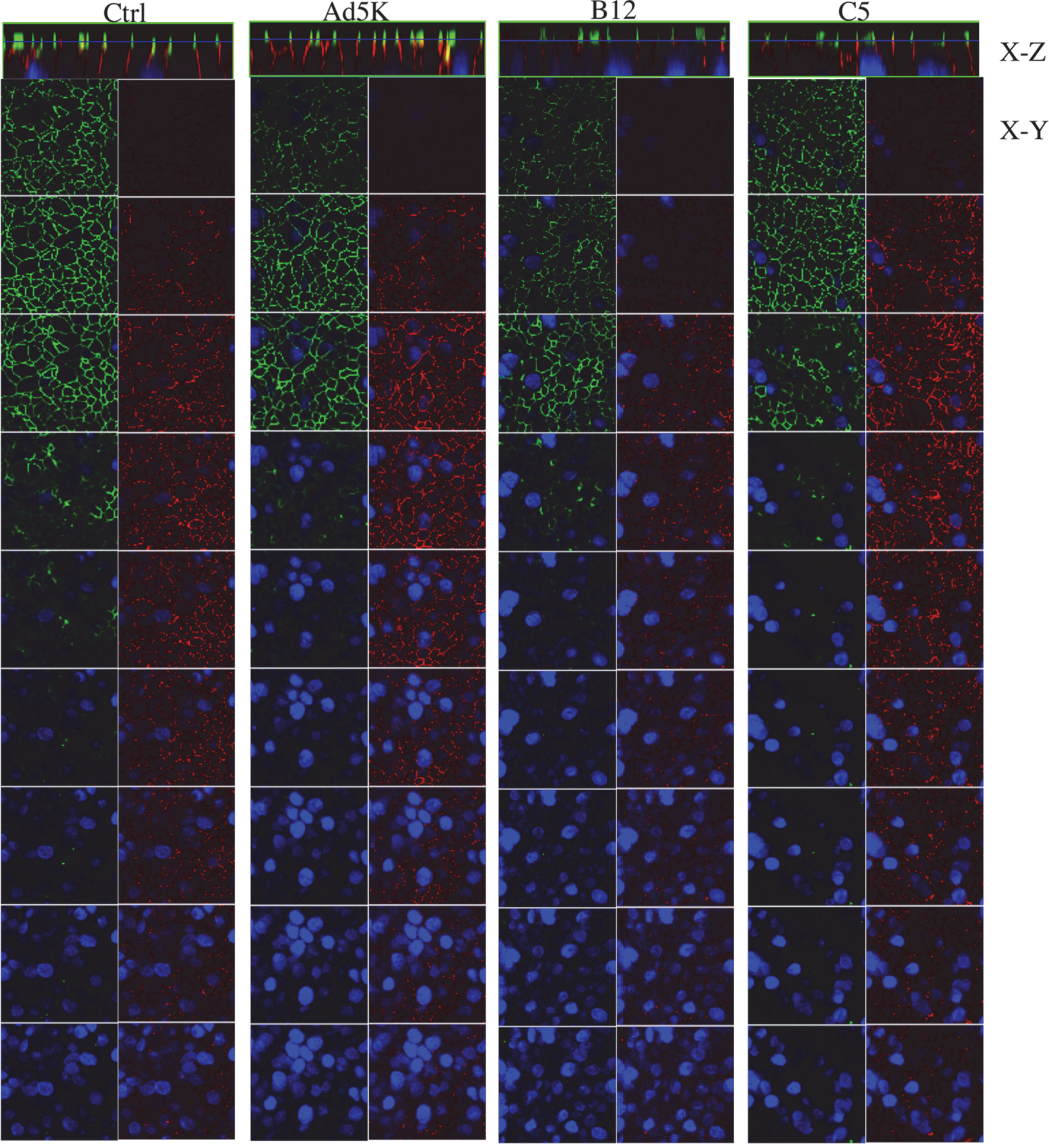
Remodeling of cell-cell junction between T84 cells by penton base-containing fiber complexes. T84 cells cultured on the insert of transwell with well developed TJs were basolateraly treated with media (Ctrl), Ad5 fiber knob (Ad5K, 100 ng/μl in fresh media), B12 or C5 fractions (5x diluted in fresh media) for 6 hr. Cells were then fixed, permeabilized and double stained for CAR and DSG-2. The localization of CAR (green) and DGS-2 (red) staining in consecutive optical sections taken from top to a depth of 5 μm below the apical surface is depicted. Individual optical sections show weak staining for both CAR and DSG-2 following incubation with B12 fraction. Cells were visualized using Zeiss confocal microscopy LSM510. The nuclei were stained with DAPI using Vectachield mounting medium. Data shown are representative of the results with the fractions from three independent gel filtration chromatography experiments.

**Fig 8:**
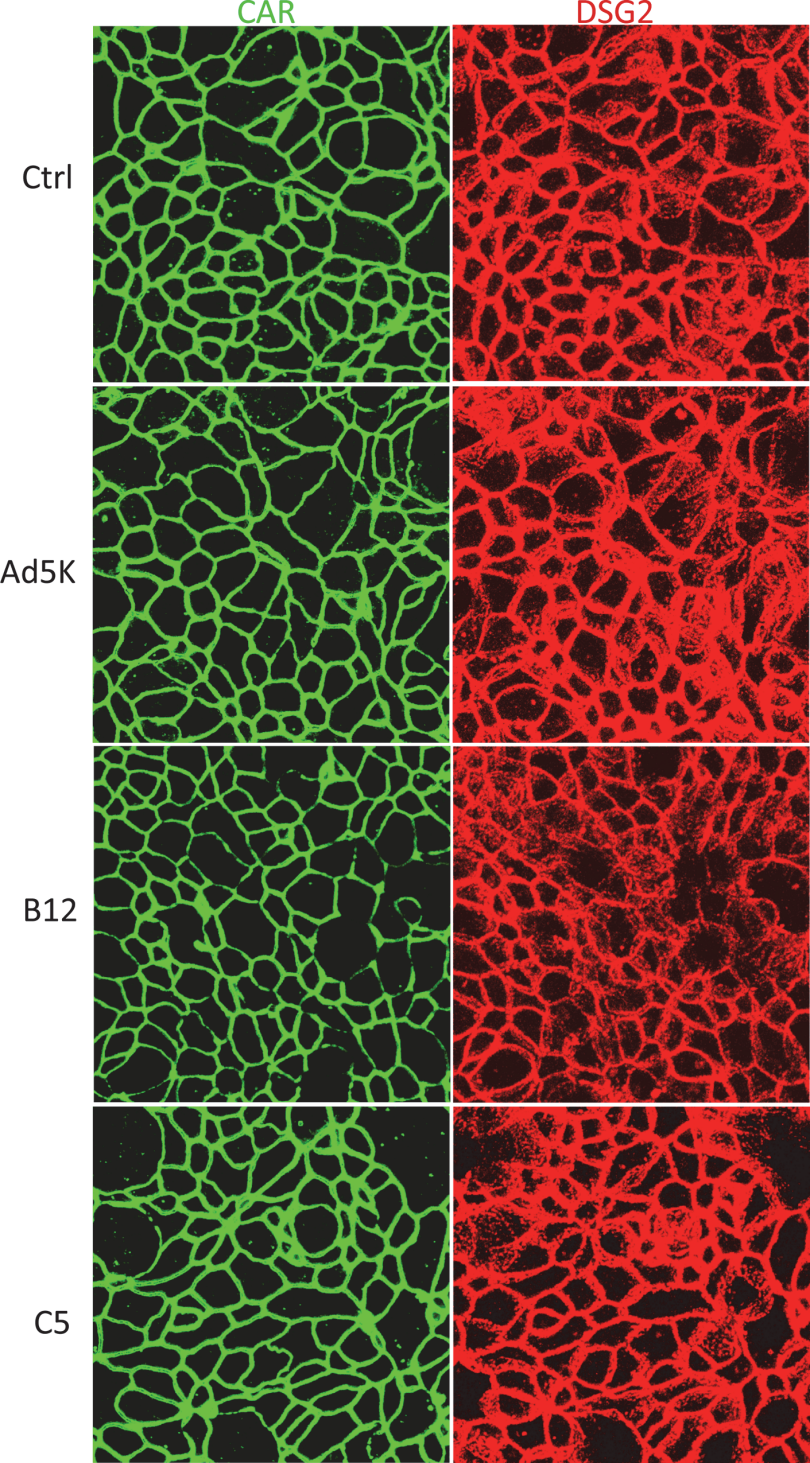
No detectable reduction in the expression of CAR and DSG-2 following treatment with B12 fraction. Images of merged optical sections of the CAR and DSG-2 stainings shown in Fig. 7 are shown. No obvious reduction in the staining of CAR and DSG-2 following treatment with B12 fraction was detected.

Finally, we compared the capacity of the two types of fiber complexes in remodeling cell-cell junction between T84 cells. T84 cells grown on inserts of transwell were basolaterally treated with B12 or C5 fractions, or recombinant Ad5 fiber knob (Ad5K). Cells were co-stained for CAR and DSG-2. Data from consecutive optical sections from the apical surface downwards show that the staining of CAR (green) and DSG-2 (red) became significantly weaker following incubation with B12 fraction, whereas incubation with fraction C5 or recombinant Ad5 fiber knob did not cause detectable changes in CAR and DSG-2 staining pattern (Fig. 7). To assess the probability of down-regulated expression of CAR and DSG-2 following treatment with B12 fraction, we merged the entire set of optical slices for CAR and DSG-2 staining (Fig. 8), the merged images indicate no obvious reduction in the CAR and DSG-2 staining intensity following B12 or C5 treatment compared to the control, suggesting the weaker staining of CAR and DSG-2 in the optical sections shown in Fig. 7 was unlikely due to down regulated expression of CAR and DSG-2, but due to junctional remodeling.

## Discussion

Our findings show that although few cells in the polarized T84 cell cultures were initially infected by Ad5, cell-cell junctions became progressively distorted during the progression of infection. This was associated with the release of fiber from the infected cells. Treatment of T84 cells with CM from Ad5 infected culture simulated the distortion of cell-cell junctions at the early stage of Ad5 infection. In consistency with previous reports on the effect of receptor masking [16], treatment of T84 cells with CM from Ad5 infected culture was associated with a reduced infectivity of Ad5-GFP. However, the infectivity of Ad5F35-GFP vector was potentiated, suggesting that distortion of cell-cell junctions increased the accessibility of the receptor molecules that were otherwise inaccessible to adenoviruses. Furthermore, our studies identified two types of fiber containing complexes in the virion-free supernatant of Ad5 infected cultures: the complexes containing fiber, penton base and hexon and the complexes predominantly containing fiber and hexon. Our LC-MS/MS analyses suggest that these complexes did not contain minor structural proteins such as protein VIII and IIIa. Instead, relatively high concentration of adenoviral 100 kDa protein, which plays crucial role in hexon folding and trimerization [23] were found in both B12 and C5 fraction. Whereas the penton base-containing fiber complexes can penetrate into cells and induce distortion of cell-cell junctions, binding of the fiber complexes without penton base to cell surface did not cause apparent intracellular internalization, no apparent effect on the distortion of cell-cell junction was observed.

Excessive production of structural proteins in relation to the viral particle assembly is common in Ad infection [15–17,25]. Virion-like particle dodecahedra consisting of penton base and fiber are generated in infected cells during the infection of Ad serotypes 3, 7, 11 and 14 that utilize DSG-2 as a cellular receptor [5,26]. Dodecahedra can also be formed by overexpression of penton base alone or in combination with fiber [18]. The 3.8 Å crystal structure of Ad3 penton base dodecamer has shown that the dodecahedric structure is stabilized by N-terminal strand-swapping between neighboring penton base molecules. This event is controlled by both residues 59–61 involved in putative salt bridges between pentamers, and by the disordered N-terminal residues 1–47 [27]. Compared to dodecahedra containing only penton base, dodecahedra containing both penton base and fiber molecules can more efficiently penetrate into host cells [21]. Our findings show that structural proteins unincorporated into viral particles during Ad5 infection form two types of complexes containing fiber, penton base and hexon molecules, or containing predominantly fiber and hexon molecules. The detection of penton base molecules in the C5 fraction using LC-MS/MS analysis was likely caused by a contamination in the fraction, because during the gel filtration preparation process, the C5 fraction was collected after the B12 fraction. However, penton base molecules in the C5 fraction were unlikely present in the complex, this is supported by the findings that the binding of penton base to cell surface was not detected in the flow cytometry analysis following incubation of fraction C5 with A549 cells (Fig. 4C), and fiber molecules were not internalized following incubation of fraction C5 with T84 cells (Fig. 5 and Fig. 6). Whereas the penton base-containing fiber complexes penetrated into host cells, fiber complexes without penton base did not internalize into host cells following binding to cell surface receptors.

The presence of hexon-containing fiber complexes was not reported in previous studies. Although the mechanisms for the formation of the fiber-containing complexes in Ad5 infection are currently unknown, they are probably formed as intermediate products towards the assembly of adenoviral capsid. This is supported by the findings in the LC-MS/MS analysis that the complexes in B12 and C5 fractions both contain the adenoviral 100 kDa protein, which is required for the folding and trimerization of hexon protein [23]. The present investigation focused on the fiber-containing complexes released from the initial round(s) of Ad5 infection, further investigation is needed to clarify whether these fiber-containing complexes are secreted prior to cell lysis.

Previous studies have reported that fiber molecules can activate the ERK and NF-kB signaling, and induce transcription of inflammatory genes [28], cytokines can impair the integrity and function of tight junction [29]. Our data suggest that cytokines are unlikely causative for the effects we observed here. We used gel filtration chromatography to fractionate the supernatant, the protein components in B12 and C5 fraction are much larger than the cytokines, which are in the range of 5 to 20 kDa. This coincides with the absence of cytokine peptides in the LC-MS/MS analysis.

Nevertheless, our data indicate that in the virion-free CM of Ad5 infected culture, the majority of the fiber molecules residue in the complexes with other adenoviral structural proteins, and fiber molecules in complexes with low content of penton base or free fiber molecules did not show intracellular internalization. Apparent distortion of CAR and DSG-2 staining pattern was observed following treatment of T84 cells with supernatant fractions containing fiber molecules, penton base and hexon, but not with fractions without penton base, or with Ad5 knob. These findings appear to be inconsistent with previous study suggesting that free fiber molecules would out-compete the CAR-CAR binding between the epithelial cells and thereby increase the epithelial permeability [22]. In consistency with the role of penton base in mediating internalization of adenoviral particles [7], our findings indicate that in the absence of sufficient amount of penton base, Ad5 fiber binding to CAR would not be sufficient to induce internalization of fiber containing complexes, internalization of fiber and penton base was associated with remodeling of cell-cell junctions as demonstrated by the staining patterns of CAR and DSG-2.

## Materials and Methods

### Cells and adenovirus used in this study

A549 cells were cultured in Dulbecco's modified essential medium (DMEM, Invitrogen) supplemented with 10% heat-inactivated fetal calf serum (FCS), 100 U/ml penicillin, and 100 μg/ml streptomycin (1% pen/strep). T84 cells (ATCC, CCL-248) were maintained and grown in DMEM-Ham’s F-12 (DMEM-F12; 1:1) medium containing 10% FCS, 1% pen/strep and 1% L-glutamine (Invitrogen). Cells were routinely passaged at 50–75% confluence. To achieve cell polarization, cells were grown in the same medium and seeded at 2 x 10^5^ cells/100 μl on 6.5-mm Transwell filters (clear polystyrene, 3 μm pore size; Corning). T84 cells were maintained by changing fresh media every two days till transepithelial electrical resistance reading was stably above 3 000 Ω cm^2^, measured with epithelial resistant meter ESR-II (millipore). The T84 cultures were then infected by wild type Ad5 from the apical surface at 20 pfu/cell. The unbound viruses were washed away with the DMEM-F12 media at 4 hpi.

Ad5-green fluorescent protein (GFP) is an Ad5 vector encoding a GFP expression cassette driven by CMV promoter in the E1 region. Ad5F35-GFP is an Ad5-GFP based vector with the knob and the 3´ part of the shaft domain switched to those of Ad35 [30]. Wild type Ad5 was propagated in A549 cells, and the GFP encoding viruses were propagated in 293 cells. Virus purification and titration were performed as described previously [16].

### Preparation of virion-free fiber containing supernatant

A549 cells were infected with wild type Ad5 at a MOI of 1 for 4 hrs. Subsequently, cells were maintained in fresh medium containing 2% FCS for 3 days. Supernatants were collected from mock infection or Ad5 infected cultures and spun at 1 000 g for 5 min to remove floating cells. To remove viral particles, supernatant was spun twice at 108 000 g for 1 hr at 4°C. The upper part (1 cm above the bottom) of supernatant was collected and concentrated by ultrafiltration spin filter (Pall) with a cut-off of 10 kilo dalton. After filtration using a 0.22 μM sterile filter, the virion-free supernatant was stored at -80°C in 1 ml aliquots until use. Viral infection was not observed in A549 cell cultures following incubation with such supernatant for one week. Before use, the supernatant was diluted 1:10 in fresh medium without FCS. To standardize the conditioned media from different preparation, the amount of functional fiber molecules in the media were measured by infection inhibition assay. Two hundred μl of diluted media contained about 8 functional units of fiber molecules (see below).

### Fractionation of viral structural proteins in gel filtration chromatography

The virion-free fiber containing supernatant prepared as described above was fractionated using gel filtration chromatography with an ÄKTA purifier liquid-chromatography system, using a prepacked Superdex 200 preparation grade column (HiLoad 16/60, GE, Sweden). One milliliter of the concentrated supernatant was loaded onto the column. Chromatography was carried out at 4°C in 0.05 M sodium phosphate buffer (pH 7.4) with 0.15 M NaCl at a flow rate of 0.5 ml/min. The elution was monitored by absorbance at 280 nm and fractions of 2 ml, named according to their positions at the collection rack, were collected.

### Western-blot detection of structural proteins and functional testing of fiber containing fractions

We first used western-blot to detect fiber and penton base in the fractions representing different protein peaks from gel filtration chromatography. Twenty μl aliquots of fractions were separated on 10% SDS-PAGE gel and subsequently transferred to PVDF membrane. The membrane was first incubated with 7.5% nonfat milk powder in PBS, then probed with primary antibodies at pre-determined dilutions, followed by washing and probing with HRP-conjugated secondary antibodies. The specific protein bands were visualized using an ECL kit (GE Health Care).

The fractions with high fiber content were further analyzed in flow cytometry analysis for assessing cell surface binding of structural proteins. In brief, 2 x 10^5^ A549 cells were suspended in PBS containing 2% fetal calf serum (FCS) and subsequently incubated with 200 μl of the selected protein fractions for 15 min on ice. Cells were then washed once with PBS containing 2% FCS and further incubated with mouse anti-fiber antibody (mAb 4D2, 1:200) and rabbit anti-penton base antibody (at 1:3000 dilution), followed by staining with Alexa Fluor 647 conjugated donkey anti-mouse IgG and Alexa Fluor 488 conjugated donkey anti-rabbit IgG. In parallel, cells incubated with different fractions were incubated with FITC conjugated goat anti-hexon antibody and mouse anti-fiber antibody followed by Alexa Fluor 647 conjugated donkey anti-mouse IgG. Finally, cells were washed and resuspended in PBS containing 1 μg/ml 7-aminoactinomycin D (7-AAD, Sigma). Living cells negatively stained with 7-AAD were then analyzed by flow cytometry (FACS Calibur, BD) for binding of Ad structural proteins to cell surface. All procedures for antibody staining were performed on ice. Protein fractions containing the same types of Ad structural proteins were combined and concentrated by a factor of 5 using ultrafiltration spin filter (Pall) with a cut-off of 10 kilo Dalton. The concentrated samples were sterile filtrated and stored at -80°C until use.

### Titration of fiber content in Ad5 CM and in fractions from gel filtration chromatography

Functional titration of fiber content in Ad5 CM and fractions from gel filtration chromatography was measured by its capacity to inhibit the infection of Ad5-GFP virus in A549 cells. A549 cells grown in 24-well plates at 1x10^5^ cells per well were first incubated with 200 μl of a serial dilution of virion-free supernatant or fractions from gel filtration chromatography at 37°C for 30 min, cells were then washed with fresh media and infected with Ad5-GFP at a MOI of 100. After one hr incubation, non-attached viruses were washed off with fresh medium. At 24 hpi, cells were harvested and measured for the percentage of GFP positive cells with a FACS Calibur. The dilution leading to 50% of inhibition of infection was defined as one functional unit of fiber content.

### Western blot analysis and LC-MS/MS characterization of the fiber containing fractions in gel filtration chromatography

To characterize the components in the fiber containing fractions of gel filtration chromatography, aliquots from different fractions were first separated on precasted 4–20% native PAGE gel (BioRad), and then transferred onto PVDF membrane. Following incubation in PBS containing 7.5% nonfat milk powder and 1% FCS for 1 hr at room temperature, and probing with anti-fiber 4D2, rabbit anti-penton base, and FITC-conjugated anti-hexon antibodies diluted in PBS containing 1%FCS for another 1 hr, the membrane was washed 4 times and subsequently probed with Alexa Fluor 568 conjugated goat anti–mouse IgG and Alexa Fluor 647 conjugated goat anti-rabbit IgG simultaneously for 1 hr at room temperature. After another 4 washing steps, the membrane was scanned with a fluorescence laser scanning system (PharosFX Plus system, Bio-Rad) using 3-laser channels (at 488 nm, 532 nm or 663 nm). Protein complexes consisting of 2 or 3 proteins can be detected as merged color bands detected in different channels. Negative control samples were supernatant from non-infected cultures. Primary antibodies used were mouse anti-fiber mAb 4D2 (1:5000, Abcam), a polyclonal rabbit anti-penton base antibody (1:5000, a gift of Dr. Saw-See Hong), and FITC conjugated goat anti hexon-antibodies (1:800, Millipore).

In parallel, the main protein band of the B12 and C5 fraction as depicted in Fig 4D was excised from the native PAGE gel following staining with SimplyBlue (Invitrogen). The proteins were digested with trypsin, the resulting peptides were separated by capillary liquid chromatography/tandam mass spectrometry (LC-MS/MS) using ESI-QUAD-TOF (Boyuan Biotech, Shanghai). The identity of the peptides was searched for in the NCBI protein database for human adenoviruses.

### Immunostaining analyses

Cells cultured in 8-well chamber glass slides (BD Falcon), or 6.5 mm transwell inserted filters (Corning) were washed once with ice-cold PBS, then fixed with 4% paraformaldehyde for 30 min at 4°C. After fixation, cells were permeabilized with 0.5% Triton X100 for 5 min at room temperature and washed with PBS three times. Following blocking with 2% FCS in PBS for 1 hr at room temperature, primary antibody staining was performed at 4°C overnight. Primary antibodies used in the assay were rabbit anti-CAR polyclonal antibody CAR72 (1:3000, [31]), mouse anti-fiber mAb (clone 4D2 1:200, GeneTex), mouse anti-DSG-2 mAb (clone 6D8, 1:40, GeneTex), FITC conjugated goat anti-hexon polyclonal antibody (1:400, Millipore) and rabbit anti-penton base polyclonal antibody (1:2000). After three washes with PBS containing 2% FCS, cells were incubated with 1:300 diluted secondary antibodies (Alexa Fluor 488 conjugated donkey anti-rabbit IgG, or Alexa Fluor 568 conjugated donkey anti-mouse IgG) for 1 hr at room temperature. Co-staining of fiber and plasma membrane marker sodium potassium ATPase (ab76020, Abcam, 1:200), and its subsequent visualization with the secondary antibodies, was performed in the same manner. Glass slides were then washed and mounted using Vectashield containing DAPI (Vector Labs). Membrane of the transwell insert were cut off and also mounted using Vectashield containing DAPI and stored at 4°C till microscopic visualization. Photographs were taken with Axio Obersver Z1 florescent microscopy (Carl Zeiss). Confocal images were taken with Zeiss META confocal laser-scanning microscope 510 or 700 using 63× oil lenses.

## Supporting Information

S1 Fig Elution profile of gel filtration chromatography with supernatant from Ad5-GFP infected A549 culture. Concentrated supernatant of A549 cultures infected with Ad5-GFP under the same conditions as infection with the wild-type Ad5 was fractionated with the Superdex 200 column as described for in the experiment in Fig. 4A. The red arrow indicates that the peaks containing large Ad structure proteins were not detected here under conditions without Ad structural protein production.
(TIF)
