# Supplementary figures and images for: Two Types of Functionally Distinct Fiber Containing Structural Protein Complexes Are Produced during Infection of Adenovirus Serotype 5
Source: PLoS One. 2015 Feb 27;10(2):e0117976. doi: 10.1371/journal.pone.0117976 (PMC4344211; doi:10.1371/journal.pone.0117976)

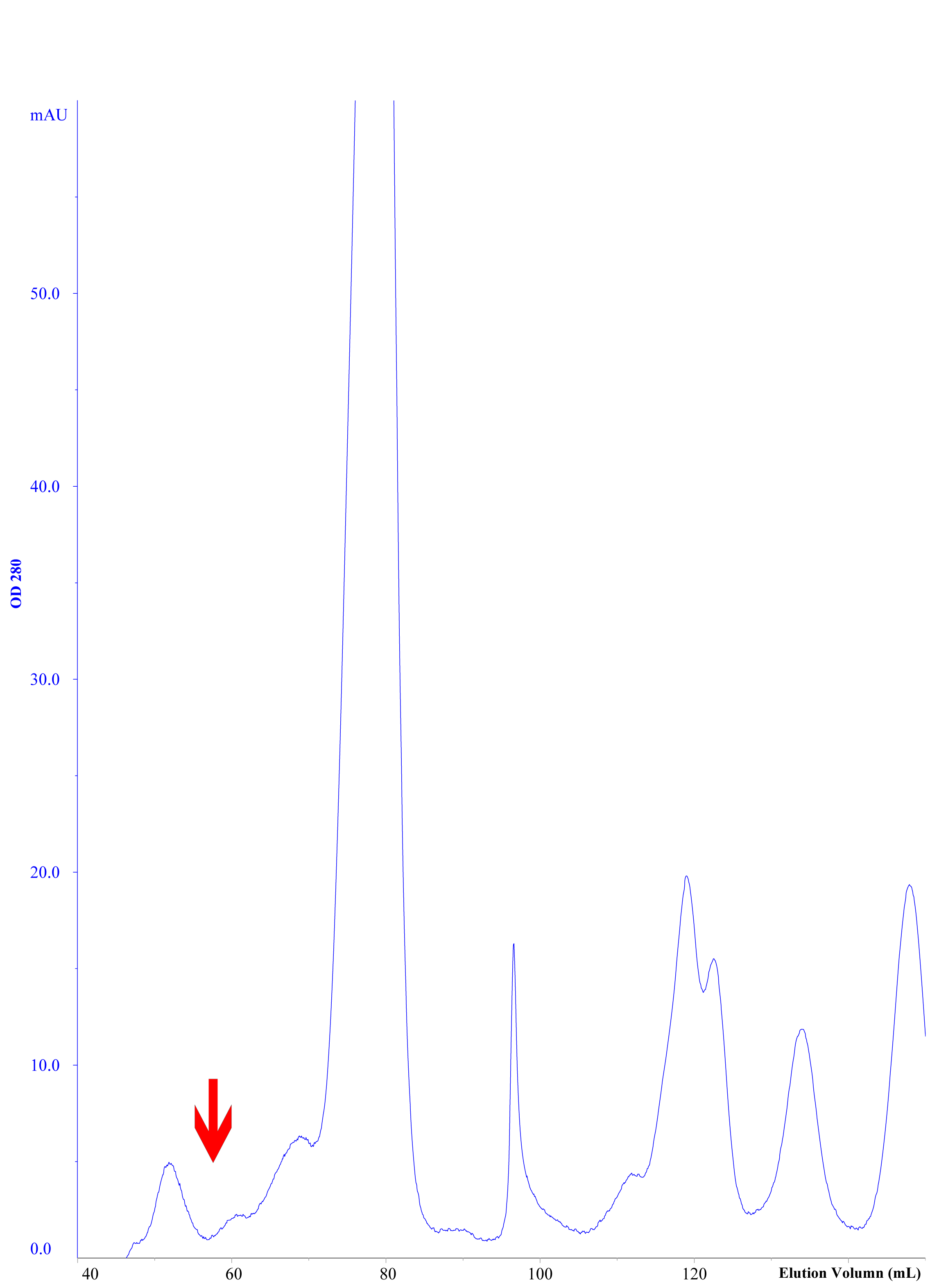

Supplement: S1 Fig — Concentrated supernatant of A549 cultures infected with Ad5-GFP under the same conditions as infection with the wild-type Ad5 was fractionated with the Superdex 200 column as described for in the experiment in Fig. 4A. The red arrow indicates that the peaks containing large Ad structure proteins were not detected here under conditions without Ad structural protein production. (TIF) [file pone.0117976.s001.tif]
